# Comparison of the effectiveness of online supportive parenting intervention (SPACE) and timid to tiger program (FTTT) on childhood anxiety disorders and family accommodation with samples of Iranian parents

**DOI:** 10.3389/fpsyg.2022.1001705

**Published:** 2022-10-20

**Authors:** Mohaddeseh Sadat Ghodrat, Asma Aghebati, Ali Asghar Asgharnejad Farid, Elham Shirazi

**Affiliations:** ^1^Department of Clinical Psychology, School of Behavioral Sciences and Mental Health, Tehran Institute of Psychiatry, Iran University of Medical Sciences, Tehran, Iran; ^2^Department of Psychiatry, School of Behavioral Sciences and Mental Health, Tehran Institute of Psychiatry, Iran University of Medical Sciences, Tehran, Iran

**Keywords:** child anxiety, anxiety, family accommodation, FTTT, SPACE

## Abstract

Studies have supported the effectiveness of the From Timid to a Tiger (FTTT) and Supportive Parenting for Anxious Childhood Emotions (SPACE) program in reducing childhood anxiety. This study is the first to compare the effectiveness of the two programs in the treatment of childhood anxiety disorder and reducing family accommodations levels. Parents of children aged 6 to 9 (*n* = 49, 49% boys) were randomly allocated to either FTTT (n 26) or SPACE (n = 23) groups, and each attended ten online sessions following the manuals of the interventions. Throughout the study, 9 participants dropped out, resulting in a total of 49 participants, and we performed statistical analyses based on data from these 49 participants. The assessment took place pre- and post-treatment and at a ten-week follow-up on parent-rated child anxiety and family accommodation measures. Analyses of variance (repeated measures) and gain scores were conducted to examine the data. The results indicated that both treatments approaches produced significant reductions in outcome measures, and the post-treatment gains of both treatments were maintained at a ten-week follow-up, though when considering the mean differences and effect sizes across the assessment scores and between groups, overall, the FTTT was significantly more effective in reducing child anxiety scores and family accommodation levels. Our results demonstrated that FTTT significantly outperformed the SPACE program in reducing childhood anxiety problems and family accommodation levels.

## Introduction

According to the *Diagnostic and Statistical Manual of Mental Disorders* (*DSM-5*, American Psychiatric Association, 2013), anxiety disorders (ADs) include disorders that share features of excessive fear and anxiety and related behavioral disturbances. The term anxiety refers to the anticipation of future threats and is associated with muscle tension and vigilance in preparation for future danger and cautious or avoidant behaviors. Under the category of anxiety disorders, *DSM-5* included seven disorders, including generalized anxiety disorder, social anxiety disorder, specific phobia, panic disorder, agoraphobia, separation anxiety disorder, and selective mutism. Also, obsessive–compulsive disorder (OCD), that was previously in the *“Anxiety Disorders”* section of *DSM-IV-TR* was added to a new chapter in *DSM-5* — *“Obsessive–Compulsive and Related Disorders.”* ADs are the most common mental disorders affecting children and adolescents, affecting 10 to 20 percent of these age groups (American Psychiatric Association, 2013). ADs have an earlier onset than other internalizing disorders among youths, and they negatively impact children’s development and functioning and place a heavy burden on parents, family members, and society (Compton et al., 2004; Kessler et al., 2005). Further, longitudinal studies have also shown that anxiety disorders in childhood could be predictors of other psychopathologies in adulthood, especially anxiety and depressive disorders and substance dependence (Pine et al., 1998). Thus, early identification and treatment of ADs are of utmost importance (e.g., Chiu et al., 2016; Ebrahimi et al., 2021).

Cognitive-behavioral therapy (CBT) is recognized as an approved treatment for childhood anxiety disorders (e.g., Nauta et al., 2003; Hudson, 2005). Notwithstanding, there are difficulties in using this treatment for some children (Monga et al., 2015). For example, CBT includes training skills to identify and challenge dysfunctional thoughts, self-regulate anxiety, and active exposure to previously avoided situations. Therefore, a prerequisite for a successful CBT intervention is the development of a therapeutic atmosphere in which the therapist and the child actively work together. However, such an atmosphere is usually not achievable in therapeutic sessions with children. In addition, many children avoid attending treatment sessions, some cannot expose anxiety-provoking situations as a part of interventions, and others do not have insight into their suffering (Lebowitz et al., 2014). In these situations, family-focused therapies could be considered an alternative treatment to in-person treatment of children. Studies show that parents play an important role in the development and maintenance of children’s anxiety problems (e.g., Nauta et al., 2003; Derisley et al., 2005; Bögels and Brechman-Toussaint, 2006). A study showed that children of parents with anxiety disorders were seven times more likely to suffer from anxiety disorders than children of parents with no mental disorders (Turner et al., 1987). According to the cognitive behavioral theory of childhood anxiety problems, some parental behavioral patterns convey anxiety-provoking cognitions and beliefs to children (e.g., a bias toward interventions; Kendall and Suveg, 2006). Creswell et al. (2010) provided a cognitive-behavioral framework that clearly demonstrates the role of parent-related factors in transmitting anxious cognitions to children. In this context, the parents’ own anxious cognitions influence their behavior which is observable as a pattern of fear reaction (e.g., parents scream when they see a spider). Similarly, parents share frightening information directly with their children (e.g., telling the child that the spider is dangerous). Parents’ anxious cognitions also affect their expectation of the child’s ability to cope with anxiety problems, leading to parents’ over-care and over-controlling behaviors, such as encouraging the child to avoid frightening situations. This way, children learn anxious cognitions resulting in the development and persistence of anxiety problems. In addition, some children avoid anxiety-provoking subjects because of the family accommodation — behaviors shown by family members that accommodate (or “give in to”) the child’s anxiety symptoms, such as providing reassurance, avoiding specific people, places or activities, or adjusting family routines (Lebowitz et al., 2013, 2014). Family accommodation is associated with increased anxiety symptoms, decrease in child’s functioning levels, and decrease in treatment effectiveness (Lebowitz et al., 2013).

To address these issues, family-focused cognitive behavioral therapies were developed as a treatment modality for childhood anxiety disorders in which both the family and the child participate in the sessions. Several studies indicated that family-centered CBT was significantly more effective than child-centered in-person CBT (Spence et al., 2000; Wood, 2006). Accordingly, several treatment programs were developed for treating childhood anxiety disorders in which only parents participate. These programs demonstrated advantages over in-person child treatment and the interventions in which parents and children participate jointly. The “From Timid to a Tiger” (FTTT) is one such treatment program, which was developed for parents of children with anxiety disorders aged 4 to 9 years old (Cartwright-Hatton, 2010). The program was designed based on the principles of CBT and behavioral parenting. FTTT is performed with parents within 10 group sessions. The program showed promising results in the treatment of children with a range of primary anxiety disorders, including separation anxiety, social anxiety, generalized anxiety, panic, agoraphobia, specific phobias, and obsessive–compulsive disorder (Cartwright-Hatton, 2010; Merry, 2011).

The Supportive Parenting for Anxious Childhood Emotions Program (SPACE) is another manualized parent-based treatment intervention that has been supported as a treatment modality for childhood anxiety disorders (e.g., Lebowitz et al., 2014, 2020; Lebowitz and Shimshoni, 2018; Lebowitz and Majdick, 2020). SPACE is developed to be used with parents of children/adolescents aged 6 to 14 years old and is exclusively parent-based, allowing for treatment delivery without the need for child collaboration. Rather than teaching parents specific sets of skills, the SPACE program aims to target the fundamental dynamics underlying the interaction between parents and anxious children (Lebowitz et al., 2014).

All in all, while these two treatment programs yielded promising results in dealing with childhood anxiety disorders, to our knowledge, no study has compared the effectiveness of the two programs in the treatment of childhood anxiety disorder. Also, prior studies on the efficacy of the two interventions were conducted in Western culture. Given the cultural differences between the Western and Eastern (e.g., Iran) cultures (e.g., Ebrahimi et al., 2021, 2022), especially in terms of parenting behavior (e.g., Darvishi et al., 2022) and connectedness between children and their families (e.g., Dwairy and Achoui, 2010), it is possible that the treatment yield different results across the two cultures, so it is essential first to study if the SPACE and FTTT programs are effective in Iranian culture. Therefore, the current study was conducted to fill these gaps in the literature. More specifically, we conducted the FTTT and SPACE programs with parents of children with a primary diagnosis of anxiety disorders and compared the two programs in terms of their effectiveness on child anxiety disorders and family accommodation levels. Of note, since this study was conducted during the COVID-19 pandemic, we decided to conduct psychotherapy sessions online to avoid the risk of infection for both the therapist and parents.

## Materials and methods

### Participants

**Figure 1:**
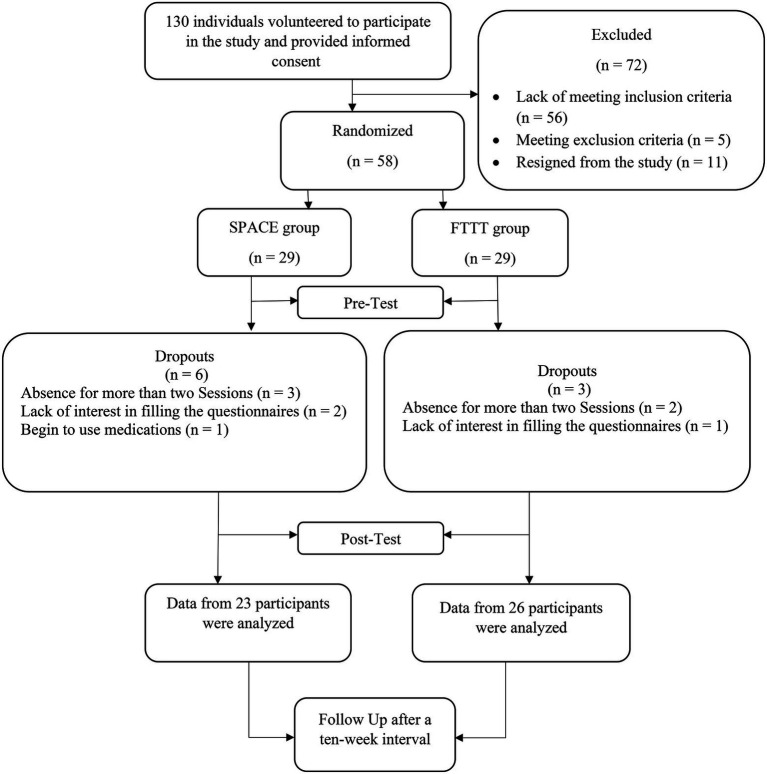
Process chart from recruitment to follow-up measurement.

A randomized pre-test, post-test, and follow-up (RPPF) design with two intervention groups was used in this study. The G-power program was used to calculate the minimum sample size needed for the study, with an alpha of 0.05, a power of 90%, and an expected medium effect size (*r* = 0.6). It was determined that a minimum of 46 participants was required to find statistically significant differences, though, considering the possibility of dropouts, we included a total of 58 individuals (29 per group). We used online advertising on social media and poster advertisements to recruit participants. 130 individuals volunteered to participate in the study. First, an independent clinical psychologist conducted the K-SADS diagnostic interview to include children with a primary diagnosis of anxiety disorders (Table 1). Consequently, parents of 58 children (aged 6–9, 49% boys) with a primary diagnosis of anxiety disorders were recruited based on the interview and inclusion/exclusion criteria. We intentionally considered this age group to be in line with the age range required by both treatment modalities. They were then randomly assigned to each intervention group based on the simple randomization method using the rand function of Excel software. Three participants from the FTTT and six participants from the SPACE group dropped out, resulting in a total of 49 participants in the FTTT (*n* = 26) and the SPACE groups (*n* = 23; A graphic depiction of the recruitment process is presented in Figure 1). Inclusion criteria consisted of (a) having a 6–9 years old child with a primary diagnosis of anxiety disorders and (b) having an education higher than a high school diploma. Exclusion criteria included (a) absence in more than two consecutive intervention sessions during the process; (b) any change in the dosage or type of medication that a child with anxiety disorders received during the interventions and follow-up period; (c) diagnosis of psychotic disorders, severe bipolar disorder, substance use disorders, and severe neurological disorders for mothers, which was assessed *via* a psychiatric interview.

**Table 1:** The psychiatric diagnoses of children in the intervention groups based on diagnostic criteria of DSM 4-TR.

	FTTT (*n*)	SPACE (*n*)
GAD+OCD	4	5
GAD+SOAD	1	1
GAD	4	3
SAD	7	5
SAD+ODD	1	–
GAD+ADHD	1	–
SOAD	2	2
SAD+GAD	4	3
GAD+OCD+MDD	1	–
GAD+OCD+Phobia	–	1
GAD+Phobia	1	1
SAD+ADHD	–	1
GAD+MDD	–	1
Total (*n*)	26	23

GAD, Generalized Anxiety Disorder; OCD, Obsessive Compulsive Disorder; SOAD, Social Anxiety Disorder; SAD, Separation Anxiety Disorder; ODD, Oppositional Defiant Disorder; ADHD, Attention Deficit and Hyperactivity Disorder; MDD, Major Depressive Disorder.

### Procedure

This study was first reviewed and approved by the Research Deputy of Iran University of Medical Sciences (Code Number = IR.IUMS.REC.1400.659). Both parents and their children provided signed informed consent after they were explained about the aims and procedure of the study and the confidentiality of the data. Next, they were asked to complete the prequestionnaires. Then, each group was divided into three groups (two groups with 10 participants and one group with 9 participants). Groups participated in online one-hour weekly sessions of FTTT or SPACE programs through a secured online program (Skyroom software) lead by the first author. Both interventions lasted for ten consecutive weeks, and both groups were asked to complete the post-questionnaires at the end of week tenth. Finally, the participants were asked to complete the questionnaires after a 10-week interval.

### Intervention

#### From timid to tiger program (FTTT program)

The From Timid to Tiger Program (FTTT; Cartwright-Hatton, 2010) is a 10-weeks CBT-based intervention for parents of children with anxiety disorders. The two primary objectives of the treatment are first to help parents supply a calm, predictable environment in which children’s behavioral difficulties are managed, and their brave, confident behavior is encouraged. Second, the group aimed to provide parents with various strategies (graded exposure, problem-solving, and behavioral experiments) to manage childhood anxiety and their own anxieties. In particular, the program focused on using fear hierarchies to help parents devise behavioral experiments to promote exposure. The content of the sessions, with a brief description, is provided in Table 2.

**Table 2:** Content of the FTTT program sessions.

Session	Content of the sessions
First	Introductions: Role of parental attention in childhood behavior; causes of anxiety disorders; introduction to cognitive behavior therapy—thoughts, feelings, and behavior and the Seven Confident Thoughts; tips on diet, caffeine, routines.
Second	Play: Building parent-–child relationship and self-esteem using child-centered play, which parents are encouraged to engage in for 5–-10 minutesmin each day
Third	Anxiety education: Fight-flight response; avoidance; Thoughts, Feelings, and Behavior in anxiety; parental modeling of anxiety
Forth	Praise and fear hierarchies: Praise for encouraging both good and brave behaviors. Tips on using praise effectively; Using fear hierarchies to tackle children’s fears.
Fifth	Rewards: Using rewards and star charts to encourage good and brave behaviors.
Sixth	Limit Setting: Using clear, calm commands to manage difficult behavior
Seventh	Ignoring: Withdrawal of attention to extinguish mild unwanted behavior and anxious reassurance seeking.
Eight	Managing Worry: listening; problem-solving; behavioral experiments; distraction; scheduled worry time.
Ninth	Using consequences and time out with an anxious child.
Tenth	Round-Up: Revision; relapse prevention; helping school to manage your child; certificates; celebration.

#### Supportive parenting for anxious childhood emotions (SPACE program)

The SPACE Program (Lebowitz and Omer, 2013) is a manualized parent-only intervention delivered during 10 to 12 weekly sessions. The treatment focuses on modifying parent behavior to help parents assume a less protective and accommodating stance toward the child and replace it with a supportive one that fosters the child’s ability to cope and self-regulate. The program attempts to lower family accommodation in eight supportive ways: (1) setting the stage, (2) charting accommodation, (3) choosing a target problem, (4) formulating a plan, (5) reducing accommodation–continued, (6) additional targets, parents take the lead, (7) additional targets–continued, (8) summary and termination. The session modules provide useful means for overcoming problems that might hinder this process: (1) teaching and modeling self-regulation, (2) coping with disruptive behavior, (3) coping with threats to self, (4) accessing support, and (5) improving collaboration between parents. The content of the sessions, with a brief description, is provided in Table 3.

**Table 3:** Content of the SPACE program sessions.

Session	Content of the sessions
First	Introductions: causes of anxiety disorders; cognitive behavioral therapy; parenting pyramid; Seven Confident Thoughts; tips on diet, caffeine, routines.
Second	Play: Building parent-–child relationship; child-centered play; building self-esteem
Third	Understanding your Child’s Anxiety: modeling; avoidance; flight-fight response
Forth	Praise and Encouragement: Effective/labeled praise; shaping brave behaviors
Fifth	Rewards: Encouraging brave behaviors through rewards and star charts
Sixth	Effective Limit Setting: Use of clear, predictive and positive commands
Seventh	Ignoring: Withdrawing attention to reduce mild unwanted behaviors
Eight	Time Out: How to use consequences for more severe unwanted behaviors
Ninth	Problem Solving/Testing Worries: revision; relapse prevention; specific examples
Tenth	Problem Solving/Testing Worries: review; future concerns; celebration; certificates

### Measures

#### Kiddie schedule for affective disorders and schizophrenia (K-SADS-PL)

The K-SADS-PL (Kaufman et al., 1997) is a semistructured diagnostic interview designed to collect information from the child/adolescent aged 6–18 and their parents. It assesses the axis I diagnoses based on the criteria of the *Diagnostic and Statistical Manual of Mental Disorders, DSM-IV*. It comprises three components: an introductory interview (demographic, health, and other background information), a screen interview (82 symptoms related to 20 diagnostic areas), and five diagnostic supplements: (1) affective disorders (major depression, dysthymia, mania, hypomania); (2) psychotic disorders; (3) anxiety disorders (social phobia, agoraphobia, specific phobia, obsessive–compulsive disorder, separation anxiety disorder, generalized anxiety disorder, panic disorder, posttraumatic stress disorder); (4) disruptive behavioral disorders (attention deficit hyperactivity disorder/ADHD, conduct disorder, oppositional defiant disorder); and (5) substance abuse, tic disorders, eating disorders, and elimination disorders (enuresis, encopresis). After interviewing the parent and child, a summary rating is made by the clinician based on all sources of information available and the use of the interviewer’s clinical judgment. Shahrivar et al. (2009) showed that the Persian K-SADS-PL enjoys acceptable psychometric properties.

#### Spence children’s anxiety scale parent-report

The Parent report version of the Spence Children’s Anxiety Scale (Spence, 1998) is a 38-item measure of childhood anxiety disorders based on *DSM-IV-TR* criteria for children aged 6–18 years. Items assess specific anxiety symptoms relating to six sub-scales: phobia, separation anxiety, panic attack/agoraphobia, obsessive–compulsive disorder, generalized anxiety disorder, and physical injury fears. Parents are asked to indicate the frequency with which each symptom occurs on a four-point scale ranging from 0 (*Never*) to 3 (*Always*). A total SCAS score is obtained by summing scores of the 38 anxiety symptom items. Persian version of the SCAS yielded acceptable psychometric properties (Mousavi et al., 2007).

#### Family accommodation scale—anxiety

FASA is a parent-report scale that assesses family accommodation within the recent month (Lebowitz et al., 2013). It includes nine questions rated on a 5-point Likert scale ranging from 0 (*never*) to 4 (*daily*). This scale evaluates the frequency of family members’ accommodation in the child’s symptoms (assessed by five items) and the change in family routines and activities (assessed by four items) in the parent. The sum of items yields a total FASA score. Persian version of the FASA yielded acceptable psychometric properties (Zamani et al., 2019).

### Data analyses

We used SPSS 20 software for data entry and statistical analyses. The normality of the distribution for outcome measures was tested using the Kolmogorov–Smirnov test, and the results supported the normality of the data (*p >* 0.05). We first analyzed the pre-test differences in demographic and outcome variables between the two groups *via* the independent *t*-tests for continuous variables and chi-squared tests for categorical variables. Results indicated that the groups did not differ significantly in terms of age, gender, child anxiety, and family accommodation scores (Tables 4–6). We analyzed the outcome measures by means of repeated measures ANOVAs, with the two treatment groups as between-subject factors and the three assessments (pre-test, post-test, and follow-up) as within-subject factors. The assumption of the sphericity of repeated-measures ANOVA was violated in both analyses (*p* < 0.05), and the epsilon (ε) value was <0.75, so we relied on the Huynh-Feldt correction when reporting the results (Girden, 1992). The following rules of thumb are used to interpret values for Partial eta squared: η^2^ = 0.01 indicates a small effect; η^2^ = 0.06 indicates a medium effect; η^2^ = 0.14 indicates a large effect. Additionally, to examine whether the decreases in the outcome variables scores from pre-test to post-test, pretest to follow up, and post-test to follow up were significantly higher for any of the groups we performed gain score analysis. This involves subtracting the pre-test scores from the post-test scores and follow up scores from the post-test and pre-test scores within each group. This creates just one independent variable with only two groups and tests whether the means of the gain scores for the two groups are equal or not. Independent-samples *t*-test is used for this analyses along with calculating Cohen’s *d* coefficients interpreted as ≤0.30 = small; 0.30–0.50 = medium; and ≥ 0.50 = strong effect sizes to examine the magnitude of gain scores differences (Cohen, 2013). It was decided beforehand that a *p* level of less than 0.05 would be accepted as indicating statistically significant results.

**Table 4:** The comparison of demographic data between intervention groups.

	Groups		Comparison	
Variables	FTTT (*n* = 26)	SPACE (*n* = 23)	*Χ*^2^	*p*
Gender(%)				
Boy	15 (57.69)	10 (43.47)	0.20	0.866
Girl	11 (42.31)	13 (56.52)		
Mother Education(%)				
Diploma	6 (23.07)	4 (17.39)	3.959	0.138
Bachelor	11(42.31)	10(43.47)		
Master & Ph.D.	9 (34.61)	9(39.13)		

**Table 5:** Descriptive statistics of anxiety and family accommodation scores in pre-test, post-test, and follow-up assessments.

Variable	Group	Pre-Test	Post-Test	Follow-up
		Mean (*SD*)	Mean (*SD*)	Mean (*SD*)
Child anxiety	FTTT	38.96 (9.16)	29.85 (7.05)	28.46 (6.69)
	SPACE	34.30 (7.58)	30.61 (7.98)	30.09 (8.11)
Family accommodation	FTTT	23.88 (8.26)	18.19 (6.65)	16.92 (6.08)
	SPACE	24.47 (7.48)	21.65 (7.53)	20.7 (6.97)

SD, Standard deviation; FTTT, From Timid to a Tiger; SPACE, Supportive Parenting for Anxious Childhood Emotions Program.

**Table 6:** Comparison of the groups based on age and baseline variables.

Variable	Group	Mean (SD)	*t*	*p*
Age	FTTT	7.64 (0.99)	0.46	0.53
	SPACE	7.83 (1.19)		
Child anxiety	FTTT	38.96 (9.16)	1.92	0.06
	SPACE	34.30 (7.58)		
Family accommodation	FTTT	23.88 (8.26)	0.26	0.79
	SPACE	24.47 (7.48)		

SD, standard deviation; FTTT, from timid to a tiger; SPACE, supportive parenting for anxious childhood emotions program.

## Results

**Figure 2:**
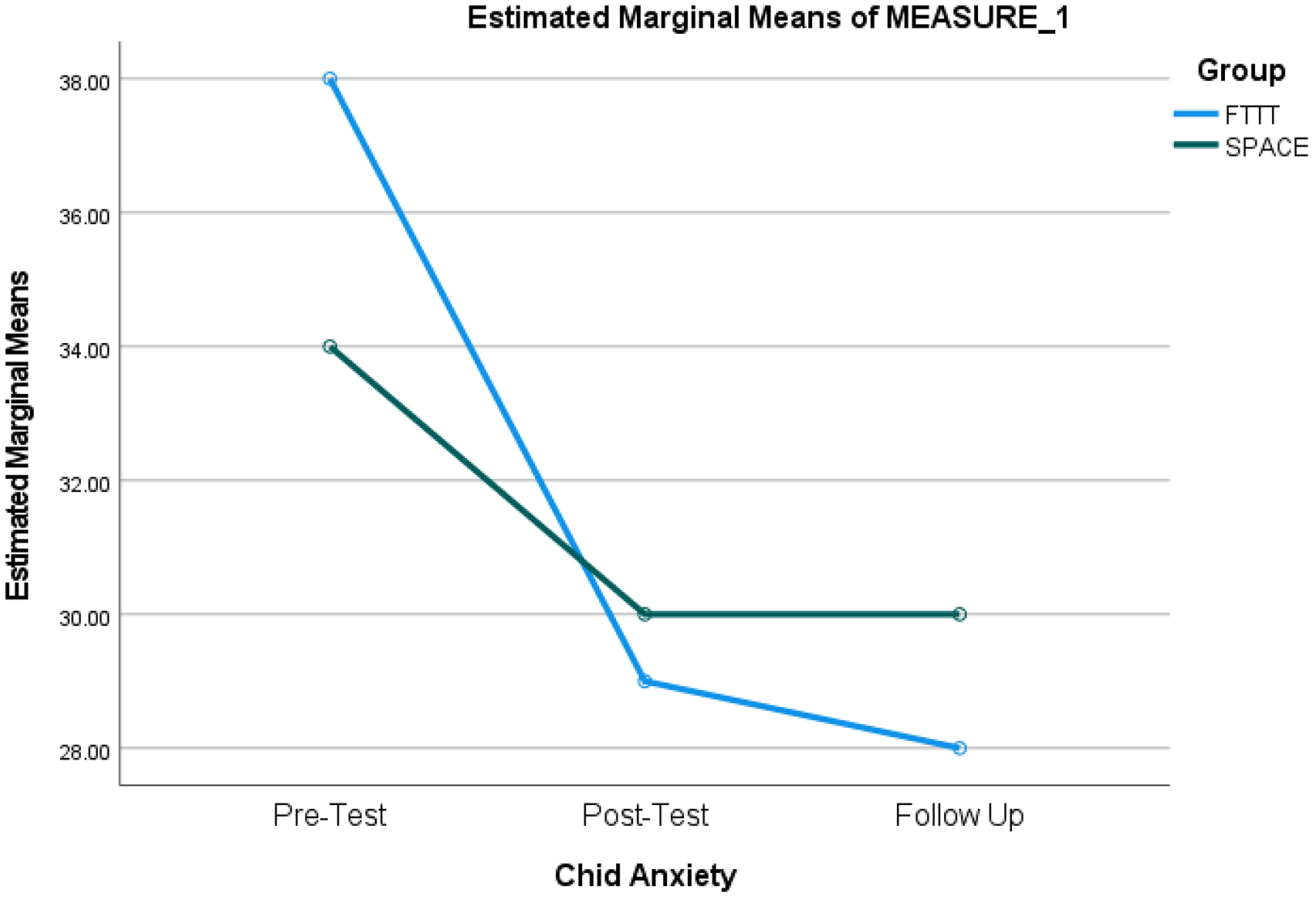
Repeated-measures ANOVA revealing significant changes from baseline to follow-up in child anxiety scores.

A repeated measures ANOVA was conducted to compare the effectiveness of FTTT and SPACE interventions on child anxiety scores. As shown in Table 7, the results demonstrated a significant main effect of time, *F*(1.30, 61) = 124.70, *p* < 0.001, *η^2^* = 0.73, indicating that there are significant differences between the assessment steps in child anxiety scores. In addition, there was a significant time × treatment interaction, *F*(1.30, 61) = 22.58, *p* < 0.001, *η^2^* = 0.32. This means that the changes in the dependent variable (i.e., child anxiety scores) across the assessment steps were statistically different between the groups. *Post hoc* paired samples *t*-tests comparisons were performed for the main effect of time and time × treatment interaction corrected with Bonferroni adjustment across the three assessment scores and separately for each group. As shown in Table 8, our results indicated significant differences between pre-test and post-test (*p* < 0.001; *d* = 1.85), pre-test and follow up (*p* < 0.001; *d* = 1.93), and post-test and follow up (*p* = 0.002; *d* = 0.66) scores for the FTTT group, while significant differences were found between pre-test and post-test (*p* < 0.001; *d* = 1.44), pre-test and follow up (*p* < 0.001; *d* = 1.67), but not post-test and follow up (*p* = 0.13) scores for the SPACE group (Figure 2).

**Table 7:** The results of repeated measures ANOVAs.

Dependent	Source		Type III sum of squares	df	Mean square	*F*	*p*	*η* ^2^
variable								
Child Anxiety	Within-subjects effect							
	Time	Sphericity Assumed	1563.58	2	781.79	124.71	0	0.73
		Greenhouse–Geisser	1563.58	1.25	1248.92	124.71	0	0.73
		Huynh-Feld	1563.58	1.3	1205.33	124.71	0	0.73
	Time × Treatment	Sphericity Assumed	283.09	2	141.55	22.58	0	0.32
		Greenhouse–Geisser	283.09	1.25	226.12	22.58	0	0.32
		Huynh-Feldt	283.09	1.3	218.23	22.58	0	0.32
	Error (Time)	Sphericity Assumed	589.28	94	6.27			
		Greenhouse–Geisser	589.28	58.84	10.01			
		Huynh-Feldt	589.28	60.97	9.67			
	Between-subject effect							
		Intercept	150,385.62	1	150,385.62	884.87	0	0.95
		Treatment	20.95	1	20.95	0.12	0.73	0
		Error	7987.71	47	169.95			
Family Accommodation	Within-subjects effect							
	Time	Sphericity Assumed	784.94	2	392.47	81.15	0	0.63
		Greenhouse–Geisser	784.94	1.41	556.23	81.15	0	0.63
	Time × Treatment	Huynh-Feld	784.94	1.47	532.93	81.15	0	0.63
		Sphericity Assumed	74.93	2	37.46	7.75	0	0.14
		Greenhouse–Geisser	74.93	1.41	53.1	7.75	0	0.14
	Error (Time)	Huynh-Feldt	74.93	1.47	50.87	7.75	0	0.14
		Sphericity Assumed	454.6	94	4.84			
		Greenhouse–Geisser	454.6	66.32	6.85			
		Huynh-Feldt	454.6	69.23	6.57			
	Between-subject effect							
		Intercept	64405.8	1	64405.8	443.09	0	0.9
		Treatment	249.16	1	249.16	1.71	0.2	0.04
		Error	6831.77	47	145.36			

**Table 8:** Paired samples *t*-tests to make *post hoc* comparisons between three assessment scores across groups.

Dependent variable	Group	Time (I)	Time (J)	Mean Difference (I–J)	SD	*t*	*p*^a^	*d*
Child anxiety	FTTP	Post-Test	Pre-Test	9.11	4.91	9.45	0.001	1.85
		Follow-up	Post-Test	1.38	2.09	3.36	0.002	0.66
			Pre-Test	10.5	5.43	9.85	0.001	1.93
	SPACE	Post-Test	Pre-Test	3.69	2.56	6.9	0.001	1.44
		Follow-up	Post-Test	0.52	1.16	2.15	0.13	–
			Pre-Test	4.21	2.52	8.02	0.001	1.67
Family accomodation	FTTP	Post-Test	Pre-Test	5.69	4	7.24	0.001	1.42
		Follow-up	Post-Test	1.26	1.34	4.82	0.001	0.94
			Pre-Test	6.96	4.62	7.67	0.001	1.5
	SPACE	Post-Test	Pre-Test	2.71	2.83	5.01	0.001	1.04
		Follow-up	Post-Test	2.33	0.96	1.97	0.18	–
			Pre-Test	2.15	3.78	8.43	0.001	1.75

a Bonferroni adjustment for multiple comparisons.

FTTT, From Timid to a Tiger; SPACE, Supportive Parenting for Anxious Childhood Emotions Program; SD, Standard Deviation; d, Cohen’s.d.

The results also showed a non-significant between subject effect of treatment, *F* (1, 47) = 0.12, *p* = 0.73, indicating that decreases in anxiety scores across the two groups were not significantly different. However, since the between-subject analysis compares a total mean score across groups, it is not a direct measure of the mean differences across groups in each assessment score. Therefore, we performed the analyses of gain scores to examine whether the decreases in the anxiety scores from pre-test to post-test, pre-test to follow-up, and post-test to follow-up were greater for any of the groups. As shown in Table 9, the decrease in anxiety scores was significantly greater for participants in the FTTT group from pre-test to post-test (*p* < 0.001; *d* = 1.36) and pre-test to follow-up (*p* < 0.001; *d* = 1.45) but not from the post-test to follow-up (*p* = 0.08).

**Table 9:** Gain score analysis *via* independent samples *t*-tests.

Dependent variable		Mean differences between groups	SD	df	*t*	*p*^a^	*d*
Child anxiety	Post-Test – Pre-Test	−5.42	4.91	38.60	−4.91	0.0	1.36
	Follow-up – Pre-Test	−6.28	2.09	36.21	−5.29	0.0	1.45
	Follow-up – Post-Test	−0.86	5.43	39.89	−1.81	0.8	–
Family accommodation	Post-Test – Pre-Test	−2.87	3.72	47	−2.89	0.1	0.83
	Follow-up – Pre-Test	−0.31	1.86	47	−0.58	5.6	–
	Follow-up – Post-Test	−6.70	9.27	47	−2.68	0.1	0.77

a Bonferroni adjustment for multiple comparisons.

SD, Standard Deviation; d, Cohen’s.d.

**Figure 3:**
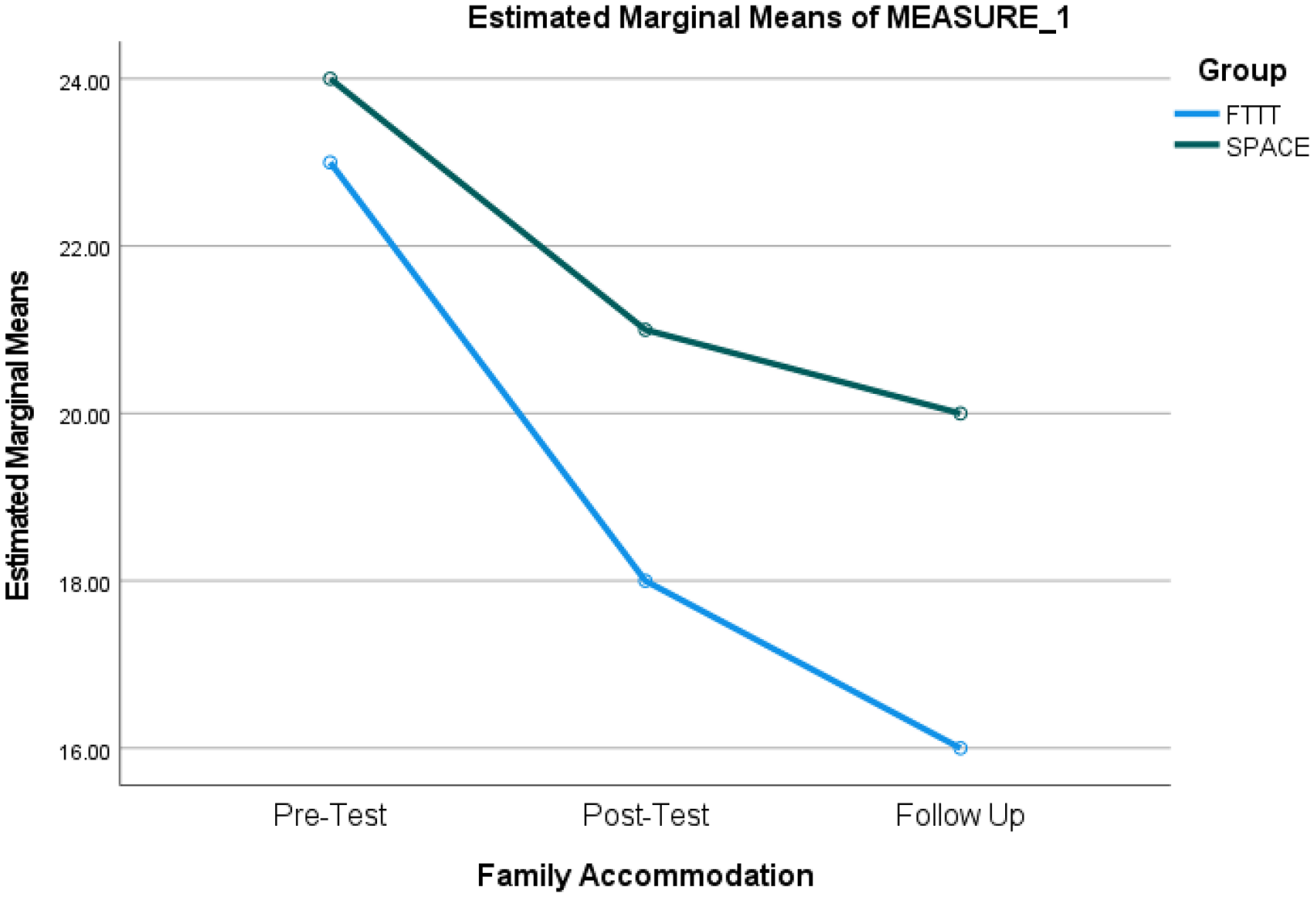
Repeated-measures ANOVA revealing significant changes from baseline to follow-up in family accommodation scores.

We conducted another repeated measures ANOVA to compare the effectiveness of FTTT and SPACE interventions on family accommodation scores. The results demonstrated a significant main effect of time, *F*(1.47, 69.22) = 81.24, *p* < 0.001, *η^2^* = 0.63, indicating significant differences between the assessment steps in family accommodation scores. In addition, there was a significant time × treatment, *F*(1.47, 69.22) = 7.75, *p* = 0.003, *η^2^* = 0.14. This means that the changes in the family accommodation score across the assessment steps are statistically different between the groups. *Post hoc* paired samples *t*-tests comparisons were performed for the main effect of time and time × treatment interaction corrected with Bonferroni adjustment across the three assessment scores and the two groups. As illustrated in Table 8, results indicated significant differences between pre-test and post-test (*p* < 0.001; *d* = 1.42), pre-test and follow-up (*p* < 0.001; *d* = 1.50), and post-test and follow-up (*p* < 0.002; *d* = 0.94) scores for the FTTT group, while significant differences were found between pre-test and post-test (*p* < 0.001; *d* = 1.04), pre-test and follow-up (*p* < 0.001; *d* = 1.75), though not post-test and follow-up (*p* = 0.18) scores for the SPACE group (Figure 3).

Additionally, the results were indicative of a non-significant between subject effect of treatment, *F*(1, 47) = 1.71, *p* = 0.20 on family accommodation, suggesting that decreases in family accommodation scores were not significantly different across the two groups. However, considering the above-explained limitation of the between-subject analysis, we performed gain scores analyses to test if decreases in the family accommodation scores from pretest to post-test, pretest to follow-up, and post-test to follow-up were greater for any of the groups. As shown in Table 9, the decrease in family accommodation scores was significantly greater for participants in the FTTT group from pre-test to post-test (*p* < 0.001; *d* = 0.83) and pre-test to follow-up (*p* < 0.001; *d* = 0.77) but not from the post-test to follow-up (*p* = 0.56).

## Discussion

While previous studies have supported the effectiveness of FTTT (Cartwright-Hatton, 2010; Merry, 2011) and SPACE (Lebowitz et al., 2014, 2020; Lebowitz and Shimshoni, 2018; Lebowitz and Majdick, 2020) programs in reducing child anxiety and family accommodation levels scores, to our knowledge, this is the first study to compare the effectiveness of the two programs in the treatment of childhood anxiety disorders and decreasing family accommodation levels. Our findings showed that both interventions substantially decreased children’s anxiety scores and family accommodation levels with substantial effect sizes. Also, the post-treatment gains of both treatments were maintained at a 10-week follow-up. The results indicated that brief treatments in this context could be highly beneficial as the participants in the present study received together only ten sessions. However, when considering the mean differences and effect sizes across the assessment scores and between groups, the results indicated that, overall, the FTTT was significantly more effective in reducing child anxiety scores and family accommodation levels. Prior studies have indicated that sometimes parents act as models of anxious behavior for their children. Parents of children with anxiety disorders are often anxious and avoid anxiety-provoking situations in the presence of their children, and this is especially true for parents who are diagnosed with anxiety disorders themselves. These behavioral patterns of parents lead to the persistence of anxiety in children. In this regard, the FTTT program helps parents learn how to control themselves in anxiety-provoking situations and prevent the emergence of emotions such as fear and anxiety, while the SPACE program does not include this aspect of child–parent relations, and this might explain the higher effectiveness of the FTTT program in reducing child anxiety scores compared to the SPACE program (Mian, 2014; Byrne et al., 2022). In the same vein, the SPACE program decreases child anxiety levels by lowering family accommodation as the main factor in developing and sustaining child anxiety, while the FTTT program is a more comprehensive intervention that attempts to improve the parent–child relationship by implementing techniques such as positive reinforcement, punishment, and management of anxious behavior; consequently, the FTTT results in higher satisfaction in parents and children and motivates individuals to continue the treatment (Mundy, 2013). Likewise, some FTTT program techniques indirectly decrease parents’ over-controlling and over-supportive behaviors, both of which are significantly associated with higher child anxiety levels. However, the SPACE program is effective only on over-supportive parental behaviors (McLeod et al., 2007; Jongerden et al., 2015).

Our results were also indicative of the higher effectiveness of the FTTT program on family accommodation than the SPACE program. To explain this finding, it could be stated that since the FTTT program reduced children’s anxiety levels significantly higher than the SPACE program, this decline might have resulted in a more significant decrease in family accommodation with children’s anxiety because the levels of children’s anxiety decreased. In contrast, in the SPACE program, parents would still have been involved in higher family accommodation levels since children’s anxiety levels decreased significantly lower than their counterparts in the FTTT group. In other words, the extent of children’s anxiety is directly associated with the levels of family accommodation.

The results from this study should be interpreted in the context of a few limitations. First, in this study, the participants included only the mothers of children. It is possible that including both parents in the intervention programs increases the effectiveness of the interventions and the alliance to the interventions. Second, all sessions were held online, so mothers who neither owned a smartphone nor had Internet access could not participate in the study. Third, because of the COVID-19 pandemic, the interventions were delivered online to avoid the risk of infection for both the therapist and parents. Therefore, we suggest future studies replicate the results through in-person sessions. Fourth, we pursued a per-protocol design, not an intention-to-treat one; the later study design type is utilized to nullify the effects of crossover and dropout, which may break the random assignment to the treatment groups in a study. Fifth, all outcomes were entirely based on parent-report data; we suggest future studies recruit data from multiple sources of information (e.g., child-report and/or independent rate outcome data). Finally, given the cultural differences between the Western and Eastern (e.g., Iran) cultures (e.g., Ebrahimi et al., 2021, 2022), especially in parenting behavior (e.g., Darvishi et al., 2022), parents in this study might have implemented the parenting techniques differently from their non-Iranian parents, so this issue could be examined in future studies.

## Conclusion and clinical implications

Our results indicated that both FTTT and SPACE programs were effective in reducing child anxiety symptoms and the levels of family accommodation, while the FTTT was significantly more effective than the SPACE program. Also, our study was the first to show the effectiveness of these treatment modalities during the COVID-19 pandemic, wherein therapists prefer online therapies over in-person interventions because of the safety of both parties. Online interventions can reduce treatment costs so more patients can benefit from them.

## Data availability statement

The raw data supporting the conclusions of this article will be made available by the authors, without undue reservation.

## Ethics statement

The studies involving human participants were reviewed and approved by the Research Deputy of Iran University of Medical Sciences. Written informed consent to participate in this study was provided by the participants’ legal guardian/next of kin.

## Author contributions

MG performed the intervention and the data analysis and prepared the manuscript. AA, AF, and ES supervised the study and reviewed and revised the manuscript. All authors have contributed to the study and agreed to the publication of the manuscript.

## Conflict of interest

The authors declare that the research was conducted in the absence of any commercial or financial relationships that could be construed as a potential conflict of interest.

## Publisher’s note

All claims expressed in this article are solely those of the authors and do not necessarily represent those of their affiliated organizations, or those of the publisher, the editors and the reviewers. Any product that may be evaluated in this article, or claim that may be made by its manufacturer, is not guaranteed or endorsed by the publisher.

## References

[ref1] American Psychiatric Association (2013). Diagnostic and Statistical Manual of Mental Disorders, 5th *Edn*. Washington, DC: American Psychiatric Pub.

[ref2] Bögels S. M. Brechman-Toussaint M. L. (2006). Family issues in child anxiety: attachment, family functioning, parental rearing and beliefs. Clin. Psychol. Rev. 26, 834–856. doi: 10.1016/j.cpr.2005.08.001, PMID: 16473441

[ref3] Byrne G. Ghráda Á. N. O’Mahony T. (2022). Parent-led cognitive behavioural therapy for children with autism spectrum conditions. A pilot study. J. Autism Dev. Disord. doi: 10.1007/s10803-022-05424-2, PMID: 35020117 PMC8753322

[ref4] Cartwright-Hatton S. (2010). From Timid to Tiger: A Treatment Manual for Parenting the Anxious Child. Chichester: John Wiley & Sons.

[ref5] Chiu A. Falk A. Walkup J. T. (2016). Anxiety disorders among children and adolescents. Focus 14, 26–33. doi: 10.1176/appi.focus.20150029, PMID: 31975791 PMC6524434

[ref6] Cohen J. (2013). Statistical Power Analysis for the Behavioral Sciences. New York, NY: Routledge.

[ref7] Compton S. N. March J. S. Brent D. Albano A. M. Weersing V. R. Curry J. (2004). Cognitive-behavioral psychotherapy for anxiety and depressive disorders in children and adolescents: an evidence-based medicine review. J. Am. Acad. Child Adolesc. Psychiatry 43, 930–959. doi: 10.1097/01.chi.0000127589.57468.bf, PMID: 15266189

[ref8] Creswell C. Cooper P. Murray L. (2010). “Intergenerational transmission of anxious information processing biases,” in Information Processing Biases and Anxiety: A Developmental Perspective. eds. Hadwin J. A. Field A. P. (Hoboken, NJ:Wiley), 279–295.

[ref9] Darvishi M. Atef Vahid M. K. Elhami Athar M. Trejos-Castillo E. Habibi Asgarabad M. (2022). The explanation of adolescent delinquent behaviors based on Jessor's problem behavior theory (PBT) in Iran: the role of individual vulnerability, opportunity risk availability, and perceived support. *Front*. Psychiatry 13:744794. doi: 10.3389/fpsyt.2022.744794, PMID: 35153871 PMC8836126

[ref10] Derisley J. Libby S. Clark S. Reynolds S. (2005). Mental health, coping and family-functioning in parents of young people with obsessive-compulsive disorder and with anxiety disorders. Br. J. Clin. Psychol. 44, 439–444. doi: 10.1348/014466505X29152, PMID: 16238888

[ref11] Dwairy M. Achoui M. (2010). Adolescents-family connectedness: A first cross-cultural research on parenting and psychological adjustment of children. J. Child Fam. Stud. 19, 8–15. doi: 10.1007/s10826-009-9335-1

[ref12] Ebrahimi A. Elhami Athar M. Bakhshizadeh M. Fathali Lavasani F. Andershed H. (2022). The Persian version of the youth psychopathic traits inventory-short version (YPI-S): A psychometric evaluation. Bull. Menn. Clin. 86, 48–66. doi: 10.1521/bumc.2022.86.1.48, PMID: 35258347

[ref13] Ebrahimi A. Elhami Athar M. Darvishi M. Colins O. F. (2021). The Persian self-report version of the antisocial process screening device (APSD-P): A psychometric evaluation [original research]. Front. Psychiatry 12:760531. doi: 10.3389/fpsyt.2021.760531, PMID: 34795601 PMC8594756

[ref14] Girden E. R. (1992). ANOVA: Repeated Measures. Newbury Park, CA: Sage Publications, Inc.

[ref15] Hudson J. L. (2005). Efficacy of cognitive—Behavioural therapy for children and adolescents with anxiety disorders. Behav. Chang. 22, 55–70. doi: 10.1375/bech.2005.22.2.55

[ref16] Jongerden L. Simon E. Bodden D. H. M. Dirksen C. D. Bögels S. M. (2015). Factors associated with the referral of anxious children to mental health care: the influence of family functioning, parenting, parental anxiety and child impairment. Int. J. Methods Psychiatr. Res. 24, 46–57. doi: 10.1002/mpr.1457, PMID: 25511424 PMC6878528

[ref17] Kaufman J. Birmaher B. Brent D. Rao U. M. A. Flynn C. Moreci P.. (1997). Schedule for affective disorders and schizophrenia for school-age children-present and lifetime version (K-SADS-PL): initial reliability and validity data. J. Am. Acad. Child Adolesc. Psychiatry 36, 980–988. doi: 10.1097/00004583-199707000-00021, PMID: 9204677

[ref18] Kendall P. C. Suveg C. (2006). “Treating anxiety disorders in youth,” in Child and Adolescent Therapy: Cognitive-Behavioral Procedures. ed. Kendall P. C. (New York, NY: The Guilford Press), 243–294.

[ref19] Kessler R. C. Berglund P. Demler O. Jin R. Merikangas K. R. Walters E. E. (2005). Lifetime prevalence and age-of-onset distributions of DSM-IV disorders in the National Comorbidity Survey Replication. Arch. Gen. Psychiatry 62, 593–602. doi: 10.1001/archpsyc.62.6.593, PMID: 15939837

[ref20] Lebowitz E. R. Majdick J. M. (2020). The SPACE program, a parent-based treatment for childhood and adolescent anxiety: clinical case illustration. J. Cogn. Psychother. 34, 107–118. doi: 10.1891/JCPSY-D-19-00028

[ref21] Lebowitz E. R. Marin C. Martino A. Shimshoni Y. Silverman W. K. (2020). Parent-based treatment as efficacious as cognitive-behavioral therapy for childhood anxiety: A randomized noninferiority study of supportive parenting for anxious childhood emotions. J. Am. Acad. Child Adolesc. Psychiatry 59, 362–372. doi: 10.1016/j.jaac.2019.02.014, PMID: 30851397 PMC6732048

[ref22] Lebowitz E. R. Omer H. (2013). Treating childhood and adolescent anxiety: A guide for caregivers. Hoboken, NJ: John Wiley & Sons.

[ref23] Lebowitz E. R. Omer H. Hermes H. Scahill L. (2014). Parent training for childhood anxiety disorders: the SPACE program. Cogn. Behav. Pract. 21, 456–469. doi: 10.1016/j.cbpra.2013.10.004

[ref24] Lebowitz E. R. Shimshoni Y. (2018). The SPACE program, a parent-based treatment for childhood and adolescent OCD: the case of jasmine. Bull. Menn. Clin. 82, 266–287. doi: 10.1521/bumc.2018.82.4.266, PMID: 30589579

[ref25] Lebowitz E. R. Woolston J. Bar-Haim Y. Calvocoressi L. Dauser C. Warnick E.. (2013). Family accommodation in pediatric anxiety disorders. Depress. Anxiety 30, 47–54. doi: 10.1002/da.21998, PMID: 22965863 PMC3932435

[ref26] McLeod B. D. Wood J. J. Weisz J. R. (2007). Examining the association between parenting and childhood anxiety: A meta-analysis. Clin. Psychol. Rev. 27, 155–172. doi: 10.1016/j.cpr.2006.09.002, PMID: 17112647

[ref27] Merry S. N. (2011). 'Timid to Tiger'group parenting training reduces anxiety diagnoses in 3-9-year-olds. Evid. Based Ment. Health 14:74. doi: 10.1136/ebmh.2011.100080, PMID: 21764873

[ref28] Mian N. D. (2014). Little children with big worries: addressing the needs of Young, anxious children and the problem of parent engagement. Clin. Child. Fam. Psychol. Rev. 17, 85–96. doi: 10.1007/s10567-013-0152-0, PMID: 23949334

[ref29] Monga S. Rosenbloom B. N. Tanha A. Owens M. Young A. (2015). Comparison of child–parent and parent-only cognitive-behavioral therapy programs for anxious children aged 5 to 7 years: short-and long-term outcomes. J. Am. Acad. Child Adolesc. Psychiatry 54, 138–146. doi: 10.1016/j.jaac.2014.10.008, PMID: 25617254

[ref30] Mousavi R. Moradi A. R. Farzad V. Mahdavi Harsini E. Spence S. H. (2007). Psychometric properties of the spence Children__AWT_QUOTE__s anxiety scale with an Iranian sample [research]. Int. J. Psychol. 1, 1–16.

[ref31] Mundy J. M. (2013). *Preliminary Examination of a Group CBT Treatment for the Parents of Young Anxious Children.*

[ref32] Nauta M. H. Scholing A. Emmelkamp P. M. Minderaa R. B. (2003). Cognitive-behavioral therapy for children with anxiety disorders in a clinical setting: no additional effect of a cognitive parent training. J. Am. Acad. Child Adolesc. Psychiatry 42, 1270–1278. doi: 10.1097/01.chi.0000085752.71002.93, PMID: 14566163

[ref33] Pine D. S. Cohen P. Gurley D. Brook J. Ma Y. (1998). The risk for early-adulthood anxiety and depressive disorders in adolescents with anxiety and depressive disorders. Arch. Gen. Psychiatry 55, 56–64. doi: 10.1001/archpsyc.55.1.56 9435761

[ref34] Shahrivar Z. Kousha M. Moallemi S. Tehrani-Doost M. Alaghband Rad J. (2009). The reliability and validity of kiddie-schedule for affective disorders and schizophrenia – present and life-time version - Persian version. Child Adolesc. Mental Health 15, 97–102. doi: 10.1111/j.1475-3588.2008.00518.x, PMID: 32847248

[ref35] Spence S. H. (1998). A measure of anxiety symptoms among children. Behav. Res. Ther. 36, 545–566. doi: 10.1016/S0005-7967(98)00034-5 9648330

[ref36] Spence S. H. Donovan C. Brechman-Toussaint M. (2000). The treatment of childhood social phobia: the effectiveness of a social skills training-based, cognitive-behavioural intervention, with and without parental involvement. J. Child Psychol. Psychiatry Allied Discip. 41, 713–726. doi: 10.1111/1469-7610.00659, PMID: 11039684

[ref37] Turner S. M. Beidel D. C. Costello A. (1987). Psychopathology in the offspring of anxiety disorders patients. J. Consult. Clin. Psychol. 55, 229–235. doi: 10.1037/0022-006X.55.2.229 3571678

[ref38] Wood J. J. (2006). Parental intrusiveness and children’s separation anxiety in a clinical sample. Child Psychiatry Hum. Dev. 37, 73–87. doi: 10.1007/s10578-006-0021-x, PMID: 16932853

[ref39] Zamani M. Jalali M. Pourahmadi E. (2019). Role of family accommodation of child symptoms, parenting style and parental stress in prediction of anxiety disorder in 6 - 10 years old children in northern Iran [original articles]. J. Gorgan Univ. Med. Sci. 21, 89–98.

